# Consumer-oriented (patient and family) outcomes from nursing in genomics: a scoping review of the literature (2012–2022)

**DOI:** 10.3389/fgene.2024.1481948

**Published:** 2024-11-29

**Authors:** Jordan N. Keels, Joanne Thomas, Kathleen A. Calzone, Laurie Badzek, Sarah Dewell, Vinaya Murthy, Rosie O’Shea, Emma T. Tonkin, Andrew A. Dwyer

**Affiliations:** ^1^ William F. Connell School of Nursing, Boston College, Chestnut Hill, MA, United States; ^2^ Genomics Policy Unit, Faculty of Life Sciences and Education, University of South Wales, Pontypridd, United Kingdom; ^3^ Global Genomics Nursing Alliance (G2NA) and National Institutes of Health, National Cancer Institute, Center for Cancer Research, Bethesda, MD, United States; ^4^ Global Genomics Nursing Alliance (G2NA) and Ross and Carol Nese College of Nursing, Penn State University, University Park, PA, United States; ^5^ Global Genomics Nursing Alliance (G2NA) and School of Nursing at Thompson Rivers University, Kamloops, BC, Canada; ^6^ Division of Medical Genetics, Department of Pediatrics, University of California San Francisco, San Francisco, CA, United States; ^7^ Cancer Genetics Service, St. James’s Hospital and Trinity College School of Medicine, Dublin, Ireland; ^8^ Global Genomics Nursing Alliance (G2NA) and Genomics Policy Unit, Faculty of Life Sciences and Education, University of South Wales, Pontypridd, United Kingdom; ^9^ Global Genomics Nursing Alliance (G2NA) and William F. Connell School of Nursing, Boston College, Chestnut Hill, MA, United States

**Keywords:** cascade screening, decision making, family history, genetic counseling, genetic testing, nursing practice, oncology nursing, precision healthcare

## Abstract

**Introduction:**

Genomics is a lifespan competency that is important for improving health outcomes for individuals, families, and communities. Nurses play a key role in genomic healthcare and realizing the potential of the genomic era.

**Methods:**

We aimed to chart the current state of genomics in nursing by conducting a systematic scoping review of the literature in four databases (2012–2022). We categorized included articles using the Cochrane Collaboration outcome domains/sub-domains and identify key topical areas.

**Results:**

Of 8532 retrieved articles, we identified 67 articles on ‘consumer-oriented outcomes’ (patient and family) for analysis. Identified articles primarily centered on themes of genetic testing and screening. Most studies reported non-interventional studies 39/67 (58%) and more than half were from the U.S.A. 34/67 (51%). Six of nine subdomains were reported on. The “patient involvement in care” subdomain was the most commonly reported subdomain (17/67, 25%) while “treatment outcomes” had the fewest reports (5/67, 8%). Overall, consumers (i.e., patients and families) had high satisfaction with nurse-led interventions.

**Discussion:**

Synthesizing findings revealed key knowledge gaps and unmet patient informational needs around genetic testing and decision support. There are opportunities for interprofessional collaboration between nursing and genetic counseling to meet the mounting demand for genomic healthcare and develop more person-centered approaches to genetic counseling and decisional support. Findings support the need for interventional studies and enhanced focus on implementation for nurses to improve consumer-oriented outcomes.

## 1 Introduction

The Human Genome Project’s initial sequencing of the human genome in 2003 marked the beginning of the “genomic era” (Collins et al., 2003). Unfolding discoveries over the past 2 decades have transformed our understanding of health and illness contributing to improved health outcomes by enabling earlier diagnosis, identifying disease risk for early intervention, and via tailored treatments (i.e., precision healthcare). Genomics is a lifespan competency with relevance across the lifespan (Calzone et al., 2013a). For example, genomics is used for preconception/prenatal testing for inherited conditions and chromosomal anomalies (i.e., aneuploidies). It is relevant for newborn screening, as well as identifying disease susceptibility. In childhood, adolescence, and adulthood, genomics is a key screening tool that informs screening recommendations, risk reduction interventions, and enables diagnosis. Moreover, genomics can aid in determining prognosis, guiding treatment decisions, and monitoring disease burden as well as disease recurrence (Calzone et al., 2013a). Genetic testing has shifted from specialty clinics (e.g., cardiac and oncology) and is now integrated into healthcare settings as another tool to inform healthcare decision making.

Early in the “genomic era”, nurses were called to be involved in genomic healthcare (Jenkins et al., 2005). Nurses are the most numerous of trained healthcare professionals with a global workforce of 29 million worldwide (Boniol et al., 2022). Nursing scope of practice spans a broad range based on academic preparation and clinical training–including advanced practice registered nurses (APRNs, e.g., nurse practitioner, nurse midwife) whose scope of practice includes assessing, diagnosing, and treating (i.e., prescriptive authority). A recent publication has reported healthcare provider-oriented outcomes (clinical and educational) related to nursing and genomics (Thomas et al., 2023) - yet consumer-oriented (patient and family) outcomes have yet to be systematically examined. With the move to improve and increase access to genomic testing, more healthcare professionals/nurses than ever before are likely to encounter patients and their families who are undergoing or have undergone genomic testing (White et al., 2020). Genetic counseling (GC) is an established discipline in the United States (United States), the United Kingdom (U.K.), and some other countries yet there are only approximately 7,000 genetic counsellors worldwide (White et al., 2020). For example, in Canada genetic counselors are largely unregulated/have no legal recognition, and there are only 484 total in Canada, which equals 1.28/100,000 population (Lambert et al., 2022). Moreover, there is unequal distribution, with 89% of the genetic counselors in Canada located in four provinces, leaving one province and two territories with zero genetic counselors (Abacan et al., 2019) In contrast, there are approximately 29 million nurses globally and nurses perform aspects of genetic counseling in many parts of the world (Lambert et al., 2022). Despite nursing’s involvement in genomic healthcare, there is little data related to outcomes from nursing involvement.

A 2012 project aiming to establish a “blueprint” for genomic nursing science (Calzone et al., 2013b) attempted to conduct a systematic review to identify and assess evidence of improved patient outcomes of care delivered by nurses with genomic competencies (i.e., “What health outcomes are associated with nursing care which incorporates genetic and genomic principles, technology and information?”) (Calzone et al., 2013b). The literature search (up to May 2012) only identified 7 of 415 (1.7%) articles meeting inclusion criteria (Calzone et al., 2013b). Thus, nearly a decade into the “genomic era”, there was insufficient evidence for a qualitative synthesis to address the question about genomic nursing outcomes. Since 2012, evidence-based applications supporting genomics in practice have grown - i.e., Clinical Pharmacogenetics Implementation Consortium (CPIC) (Relling and Klein, 2011), National Comprehensive Cancer Network (NCCN) (NCCN, 2024). Accordingly, it seems timely to re-evaluate outcomes for genomic nursing to enable nurses to use omics (i.e., genomics, proteomics, metabolomics, metagenomics, phenomics, transcriptomics) in their practice.

This study aimed to chart nursing and/or midwifery involvement in genomics (2012–2022) since the previous attempt to conduct a mixed-methods systematic review of the literature in 2012 (Calzone et al., 2013b). We conducted a systematic scoping review of the literature to identify the current state of genomics in nursing/midwifery and address the broad question “What outcomes are associated with nursing and midwifery practice that incorporates Omics research, principles, technology and information?”. Sorting identified articles according to the Cochrane Collaboration outcome taxonomy (Dodd et al., 2018). Herein, we report on consumer-oriented outcomes (2012–2022). Consumer oriented outcomes refer to outcomes that are directly relevant and meaningful to patients and their caregivers (i.e., quality of life, functionality and daily activities, adverse and side effects, etc.) (Hill). This scoping review provides a summary of the current landscape of consumer-oriented outcomes from nursing involvement in genomic healthcare and highlights future directions for nursing and genetic counseling to meet the burgeoning demand for genomic healthcare.

## 2 Methods

We conducted a systematic search of the literature and scoping review guided by the Arksey and O’Malley framework (Arksey and O'Malley, 2005; Tricco et al., 2016). The six steps of the framework include: i.) identifying the research question; ii.) identifying the relevant literature; iii.) selecting the literature; iv.) charting the data; v.) collating, summarizing, and reporting results; and vi.) synthesis of results. No human subjects were involved in this scoping review. As such, this project was exempt from ethics board review. No registered protocol is associated with this scoping review and no public or patient involvement occurred in relation to the scoping review. Covidence™ systematic review software (Covidence systematic review software, 2023) was employed for the literature search and review of identified articles. Study findings are reported using the Preferred Reporting Items for Systematic Reviews and Meta-Analyses extension for the reporting of scoping reviews (PRISMA-ScR) (Tricco et al., 2018).

### 2.1 Identifying the research question

The scoping review was guided by a single primary question “What outcomes are associated with nursing and midwifery practice that incorporates Omics research, principles, technology and information?”. For the purposes of this scoping review, nursing/midwifery practice was defined as: patient/client care, patient/client counselling, clinical interventions, health promotion, research, and education that is provided or delivered by registered nurses/midwives. Consumer-oriented outcomes are operationally defined as those outcomes that have been measured/assessed in different groups occupying different roles–i.e., members of the public, individual patients, family carers, community volunteers, or advocates (Hill). For this scoping review we focused specifically on outcomes for individual patients and families.

### 2.2 Identifying the relevant literature

With the assistance of a research librarian, we conducted literature searches (July 2022) in four databases (PubMed, CINAHL Plus, EMBASE, Web of Science core collection). The structured search used medical subject headings (MeSH) terms and key words (Supplementary Material S1).

### 2.3 Selecting the literature

Inclusion criteria for eligible studies were: i) primary research studies published in a peer reviewed journal; ii) studies reporting findings from original studies performed globally (i.e., any country of the world); iii) studies reporting results/outcomes associated with a nursing activity in Omics (i.e., genomics, proteomics, metabolomics, metagenomics, phenomics, and transcriptomics); iv) studies with an explicit focus on nursing/midwifery activities; v) published in English; vi) published since May 2012 (i.e., immediately following the publication of the original attempt at a mixed-methods systematic review) (Calzone et al., 2013b) up to July 2022. Exclusion criteria were: i) review articles, letters to the editor, or commentary articles; ii) reporting secondary or tertiary sources; iii) studies with no clear nursing/midwifery contribution; iv) studies with peripheral involvement of nurses/midwives (e.g., part of the study team); v) studies in which nursing/midwifery activities are not the study focus or without defined outcomes; vi) not published in English; vii) published prior to May 2012. Articles retrieved from the structured literature search were imported into Covidence™ (Covidence systematic review software, 2023) for screening. After removing duplicate titles, articles underwent independent, dual review of title and abstract (JT, JNK, KAC, CP, AAD, ETT). Discrepancies were determined by a third independent reviewer from the review team. Remaining articles underwent independent, dual, full-text review (JT, JNK). Any discrepancies during the review process were resolved by a third independent reviewer (KAC, AAD, ETT).

### 2.4 Charting the data

Independent investigators (JT, JNK) extracted data using a structured, predetermined data collection form. The data extraction form was developed specifically for this scoping review to capture title, authors, year, country, study population, nursing/midwife population, methods, nursing/midwife activity or intervention, genomics focus, summary of study findings/outcomes, and relevant Cochrane Collaboration outcome taxonomy (Supplementary Material S2) (Hill). In brief, the Cochrane taxonomy comprises five outcome domains (“consumer”; “healthcare provider”; “health service delivery”; “related to research”, and “societal or governmental”) each with respective sub-domains (Dodd et al., 2018). Risk of bias was not conducted due to methodological variability of included studies.

### 2.5 Collating, summarizing, and reporting results

Extracted data from the included articles were organized in a master table (Supplementary Material S3). Articles were grouped according to the Cochrane Collaboration outcome taxonomy domain ‘consumer- oriented outcomes’ that includes nine sub-domains, i.) ‘knowledge and understanding’; ii.) ‘communication’; iii.) ‘patient involvement in care process’; iv.) ‘evaluation of care’; v.) ‘support’; vi.) ‘skills acquisition’; vii.) ‘health status and wellbeing’; viii.) ‘health behavior’; and ix.) ‘treatment outcomes’. Findings are reported narratively using descriptive statistics (i.e., percentages). Results on the Cochrane ‘healthcare provider oriented outcomes’ (i.e., clinical and educational) domain have been previously reported (Thomas et al., 2023).

### 2.6 Synthesis of results

To synthesize nursing/midwifery roles in Omics within the Cochrane Collaboration ‘consumer-oriented outcomes’ domain, two investigators (JNK, AAD) reviewed and analyzed identified articles using an iterative process to identify thematic elements and map them to the sub-domains (Saunders et al., 2023). Studies were examined to chart methodologic approaches (i.e., quantitative, qualitative, mixed-methods), whether the study was interventional or non-interventional, as well as topic areas (i.e., genetic counseling, screening, oncology, rare diseases, etc.).

### 2.7 Validation

Validation is an optional step of the scoping review process that we embraced for this project. Following the data synthesis step, two genetic counselors (VM, RO) provided international perspectives (United States and Ireland) and helped interpret the data synthesis through an interprofessional lens (i.e., nursing and genetic counseling). This validation step aimed to chart future directions for interprofessional collaboration to enhance access to genomic healthcare services.

## 3 Results

**FIGURE 1:**
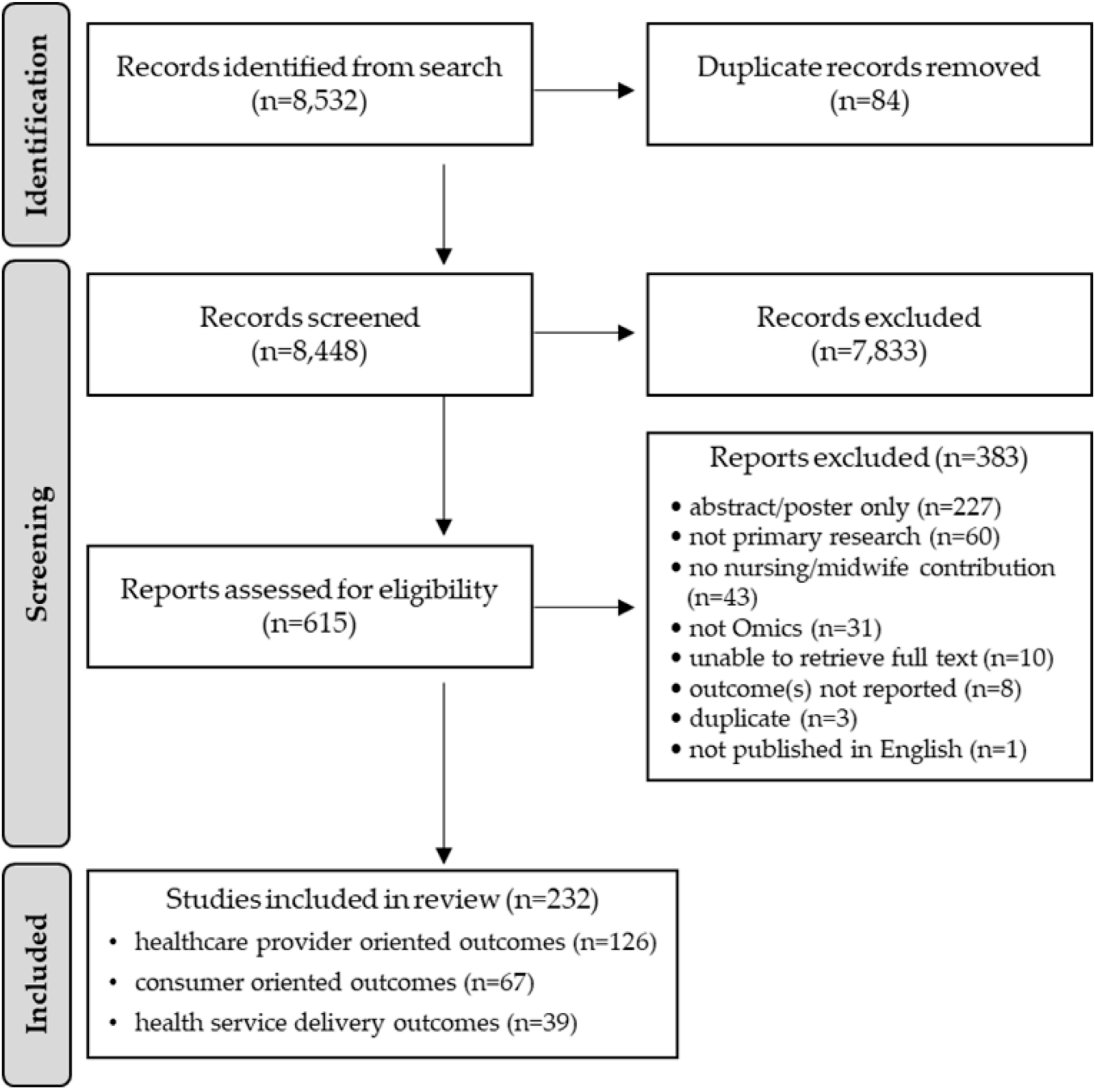
PRISMA flow diagram for the systematic scoping review (2012–2022).

The systematic, structured literature search identified 8,532 articles. After duplicates were removed 8,448 articles underwent title and abstract review. After screening, 7,833 articles were excluded, leaving 615 articles for full-text review. Full-text review yielded 232 articles for analysis (Thomas et al., 2023). The PRISMA flow diagram (Figure 1) outlines the review process and reasons for exclusion.

Overall, the 232 included studies consisted of publications from 33 different countries, primarily conducted in high income countries (Thomas et al., 2023). Included studies were classified according to the Cochrane Collaboration outcome taxonomy (Dodd et al., 2018). Three outcomes were identified: i.) “healthcare provider oriented outcomes” 126/232 (54.3%) (Thomas et al., 2023), ii.) “consumer oriented outcomes” 67/232 (28.9%), and iii.) “health service delivery outcomes 39/232 (16.8%). This article reports findings of articles relating to “consumer-oriented outcomes”. A summary table with study characteristics and key findings for included consumer oriented articles is provided in Supplementary Material.

### 3.1 Consumer-oriented outcomes

**FIGURE 2:**
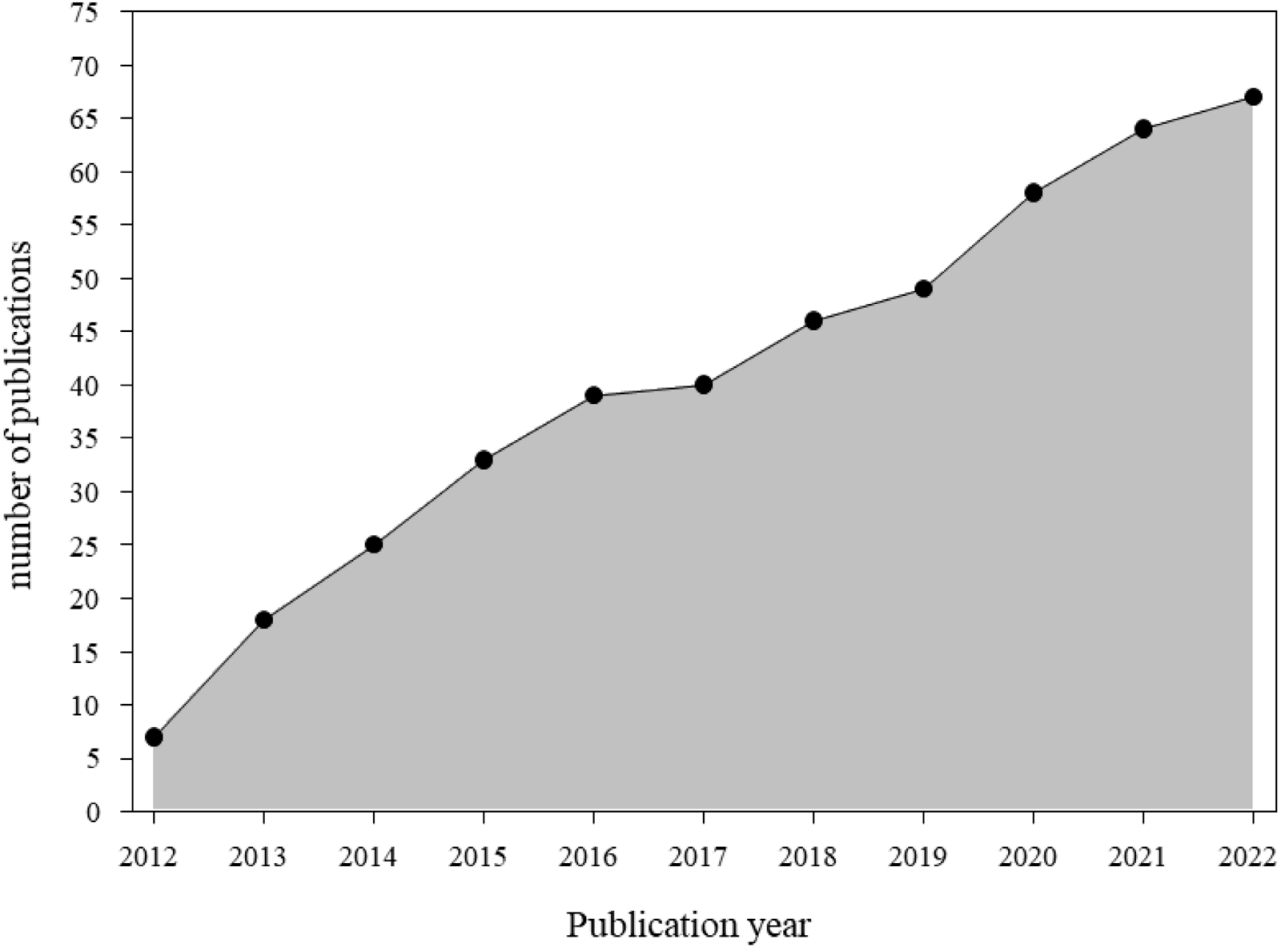
Cumulative genomic nursing publications reporting consumer-oriented outcomes by year (2012-2022: n = 67). A total of 67 articles were identified relating to consumer oriented outcomes (2012-2022). On average, 6.09±2.88 articles (median: 6) were published each year.

A total of 67 articles were identified relating to “consumer-oriented outcomes.” Articles reported on 6/9 sub-domains including: patient involvement in care process (17/66, 26%) (Labore, 2012; Jabaley et al., 2020; Paljevic, 2020; Hersperger et al., 2020; Newcomb et al., 2019; Hanish et al., 2018a; Williams et al., 2018; Li et al., 2016; Gitsels-van der Wal et al., 2015; Gitsels-van der Wal et al., 2014a; Dixon and Burton, 2014; Gitsels-van der Wal et al., 2014b; Martin et al., 2013; Cherry et al., 2013; Williams et al., 2013; Meiser et al., 2012; Chambers et al., 2016), knowledge and understanding (14/66, 21%) (Bracci et al., 2020; Waddell-Smith et al., 2016; Yeşilçinar and Güvenç, 2021; Almomani et al., 2020; Underwood and Kelber, 2015; Newcomb et al., 2014; Driessnack and Gallo, 2013; Zayts and Sarangi, 2013; Itzhaki, 2018; Silva et al., 2013; Newcomb et al., 2013; Gleeson et al., 2013; Underhill et al., 2012; Hamilton, 2012), evaluation of care (12/66, 18%) (Chandrasekaran et al., 2021; Adejumo et al., 2021; Graff et al., 2020; Murray et al., 2015; Appel and Cleiment, 2015; Oulton et al., 2021; O'Keefe et al., 2019; Rad et al., 2022; Atienza-Carrasco et al., 2020; Laws et al., 2016; Platten et al., 2012; O'Shea et al., 2012), health behavior (9/66,14%) (Katapodi et al., 2018; Jones et al., 2020; Salimzadeh et al., 2018; Arguello et al., 2018; Ingrand et al., 2016; Shahine et al., 2015; Visser et al., 2015; Kessler, 2012; Seven et al., 2017), health status and wellbeing (9/66, 14%) (Mohammed et al., 2021; Reisinho and Gomes, 2022; Jiajia et al., 2016; Withycombe et al., 2022; Anderson et al., 2021; Resnick et al., 2016; Koleck et al., 2014; Alexander et al., 2014; Voss et al., 2013), treatment outcomes (5/66, 7%) (Kashani et al., 2015; Moraes et al., 2020; White et al., 2019; Henker et al., 2016; Wesmiller et al., 2014). One article relating to consumer-oriented outcomes was considered as “other” as it did not fit into any of the nine sub-domains (Hamilton and Kopin, 2013). Articles did not report on Cochrane subdomains communication, support, and skills acquisition. Examining the publications per year (2012–2022) revealed a consistent, steady increase in nursing publications relating to “consumer oriented outcomes” with an average 6.09 ± 2.88 articles/year (median: six per year) (Figure 2).

Included articles were reported by groups from 21 different countries. American reports accounted for more than half (34/67, 51%) (Labore, 2012; Jabaley et al., 2020; Paljevic, 2020; Hersperger et al., 2020; Newcomb et al., 2019; Cherry et al., 2013; Williams et al., 2013; Underwood and Kelber, 2015; Newcomb et al., 2014; Driessnack and Gallo, 2013; Newcomb et al., 2013; Underhill et al., 2012; Graff et al., 2020; Murray et al., 2015; Appel and Cleiment, 2015; O'Keefe et al., 2019; Rad et al., 2022; Katapodi et al., 2018; Jones et al., 2020; Arguello et al., 2018; Kessler, 2012; Withycombe et al., 2022; Anderson et al., 2021; Resnick et al., 2016; Koleck et al., 2014; Alexander et al., 2014; Voss et al., 2013; Kashani et al., 2015; White et al., 2019; Henker et al., 2016; Wesmiller et al., 2014; Hamilton and Kopin, 2013; Cohen and McIlvried, 2013; Hanish et al., 2018b) of included articles followed by the Netherlands (5/67, 7%) (Gitsels-van der Wal et al., 2015; Gitsels-van der Wal et al., 2014a; Gitsels-van der Wal et al., 2014b; Martin et al., 2013; Visser et al., 2015), U.K. (3/67, 4%) (Williams et al., 2018; Chandrasekaran et al., 2021; Oulton et al., 2021), and Australia (3/67, 4%) (Meiser et al., 2012; Gleeson et al., 2013; Laws et al., 2016). The other 17 countries accounted for the remaining 22 articles, with individual countries each contributing <2% of total articles on consumer-oriented outcomes. In terms of methodology, more than half of studies were quantitative (36/67, 54%) (Gitsels-van der Wal et al., 2014a; Dixon and Burton, 2014; Martin et al., 2013; Bracci et al., 2020; Waddell-Smith et al., 2016; Yeşilçinar and Güvenç, 2021; Almomani et al., 2020; Underwood and Kelber, 2015; Newcomb et al., 2013; Chandrasekaran et al., 2021; Adejumo et al., 2021; Graff et al., 2020; Appel and Cleiment, 2015; Rad et al., 2022; Platten et al., 2012; O'Shea et al., 2012; Katapodi et al., 2018; Jones et al., 2020; Salimzadeh et al., 2018; Arguello et al., 2018; Ingrand et al., 2016; Shahine et al., 2015; Visser et al., 2015; Kessler, 2012; Seven et al., 2017; Mohammed et al., 2021; Withycombe et al., 2022; Anderson et al., 2021; Resnick et al., 2016; Koleck et al., 2014; Voss et al., 2013; Kashani et al., 2015; Moraes et al., 2020; White et al., 2019; Wesmiller et al., 2014; Cohen and McIlvried, 2013) followed by qualitative (19/67, 28%) (Labore, 2012; Paljevic, 2020; Hersperger et al., 2020; Williams et al., 2018; Li et al., 2016; Gitsels-van der Wal et al., 2015; Gitsels-van der Wal et al., 2014b; Cherry et al., 2013; Williams et al., 2013; Meiser et al., 2012; Driessnack and Gallo, 2013; Zayts and Sarangi, 2013; Gleeson et al., 2013; Underhill et al., 2012; Hamilton, 2012; Oulton et al., 2021; Atienza-Carrasco et al., 2020; Reisinho and Gomes, 2022; Hanish et al., 2018b). Less commonly employed approaches include descriptive (5/67, 7%) (Newcomb et al., 2019; Silva et al., 2013; Murray et al., 2015; Alexander et al., 2014; Henker et al., 2016), mixed-methods (4/67, 6%) (Newcomb et al., 2014; Itzhaki, 2018; Laws et al., 2016; Jiajia et al., 2016), and other (3/67, 5%) (Jabaley et al., 2020; O'Keefe et al., 2019; Hamilton and Kopin, 2013) (i.e., tool or theory development). The majority of studies were non-interventional (39/67, 58%) (Labore, 2012; Paljevic, 2020; Hersperger et al., 2020; Newcomb et al., 2019; Williams et al., 2018; Li et al., 2016; Gitsels-van der Wal et al., 2015; Gitsels-van der Wal et al., 2014a; Dixon and Burton, 2014; Gitsels-van der Wal et al., 2014b; Cherry et al., 2013; Williams et al., 2013; Almomani et al., 2020; Underwood and Kelber, 2015; Driessnack and Gallo, 2013; Zayts and Sarangi, 2013; Itzhaki, 2018; Gleeson et al., 2013; Underhill et al., 2012; Hamilton, 2012; Graff et al., 2020; Oulton et al., 2021; Atienza-Carrasco et al., 2020; Laws et al., 2016; O'Shea et al., 2012; Seven et al., 2017; Reisinho and Gomes, 2022; Jiajia et al., 2016; Withycombe et al., 2022; Anderson et al., 2021; Resnick et al., 2016; Koleck et al., 2014; Alexander et al., 2014; Voss et al., 2013; Moraes et al., 2020; White et al., 2019; Henker et al., 2016; Wesmiller et al., 2014; Hanish et al., 2018b) while 25/67 (37%) (Jabaley et al., 2020; Martin et al., 2013; Meiser et al., 2012; Bracci et al., 2020; Waddell-Smith et al., 2016; Yeşilçinar and Güvenç, 2021; Newcomb et al., 2014; Silva et al., 2013; Newcomb et al., 2013; Chandrasekaran et al., 2021; Adejumo et al., 2021; Rad et al., 2022; Platten et al., 2012; Katapodi et al., 2018; Jones et al., 2020; Salimzadeh et al., 2018; Arguello et al., 2018; Ingrand et al., 2016; Shahine et al., 2015; Visser et al., 2015; Kessler, 2012; Mohammed et al., 2021; Kashani et al., 2015; Cohen and McIlvried, 2013) were interventional studies. The remaining studies (3/67, 4%) (Murray et al., 2015; O'Keefe et al., 2019; Hamilton and Kopin, 2013) were classified as other as they related to tool or theory development.

**FIGURE 3:**
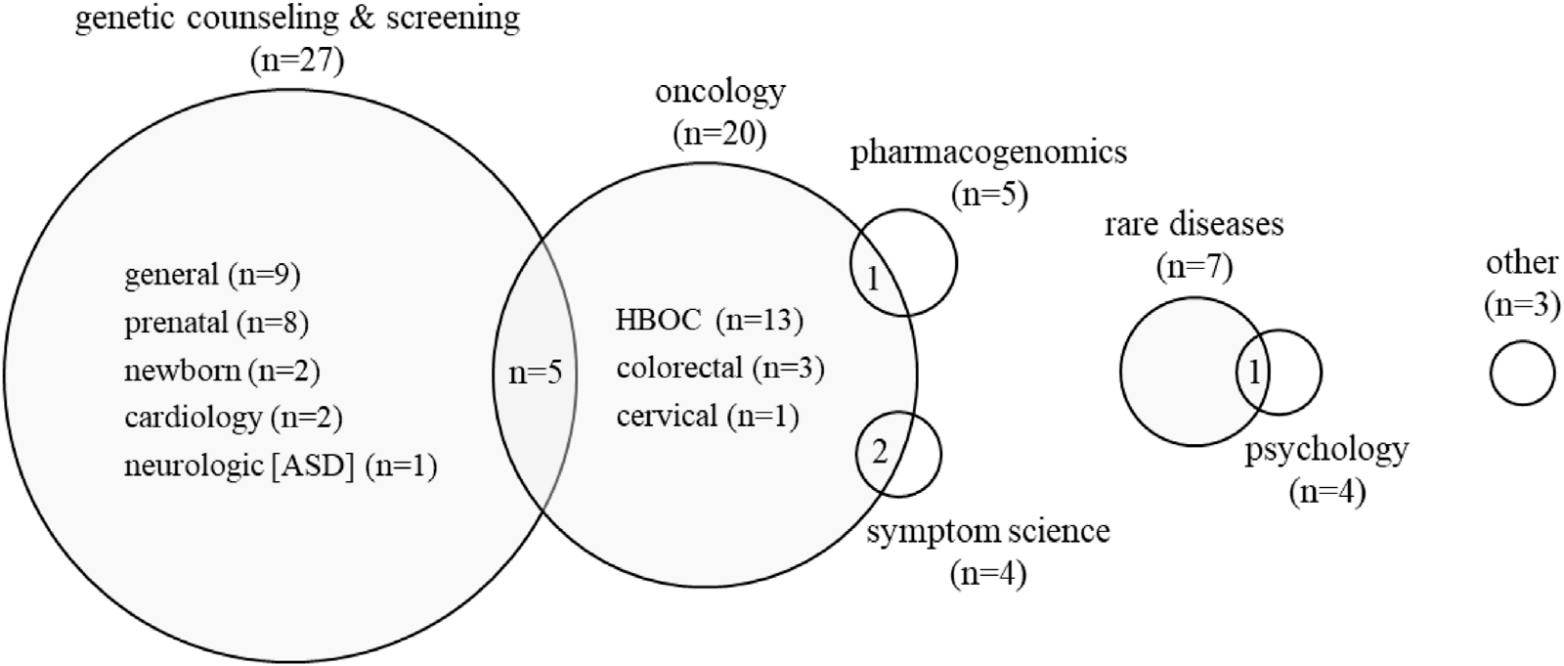
Topical areas of articles reporting consumer-oriented outcomes. The majority of articles 47/67 (70%) reported findings in the areas of genetic counseling and oncology. Circles are proportional to the number of published articles. The “Psychology” category refers to psychosocial support. “Other” category includes theory, biobanking, and pathogen genomics. ASD: autism spectrum disorder, HBOC: hereditary breast and ovarian cancer.

Thematic analysis of 67 publications on “consumer oriented outcomes” identified seven areas of focus with some overlapping topics (Figure 3). The majority of articles 47/67 (70%) reported findings in either “genetic counseling and screening” (27/67, 40%) (Paljevic, 2020; Hersperger et al., 2020; Newcomb et al., 2019; Hanish et al., 2018a; Li et al., 2016; Gitsels-van der Wal et al., 2015; Gitsels-van der Wal et al., 2014a; Dixon and Burton, 2014; Gitsels-van der Wal et al., 2014b; Martin et al., 2013; Meiser et al., 2012; Chambers et al., 2016; Bracci et al., 2020; Waddell-Smith et al., 2016; Yeşilçinar and Güvenç, 2021; Almomani et al., 2020; Underwood and Kelber, 2015; Zayts and Sarangi, 2013; Silva et al., 2013; Adejumo et al., 2021; Graff et al., 2020; Atienza-Carrasco et al., 2020; Platten et al., 2012; Arguello et al., 2018; Seven et al., 2017; Anderson et al., 2021; Kashani et al., 2015) or oncology (20/67, 30%) (Jabaley et al., 2020; Hersperger et al., 2020; Cherry et al., 2013; Chambers et al., 2016; Bracci et al., 2020; Itzhaki, 2018; Underhill et al., 2012; Hamilton, 2012; Chandrasekaran et al., 2021; Adejumo et al., 2021; Graff et al., 2020; Katapodi et al., 2018; Jones et al., 2020; Salimzadeh et al., 2018; Ingrand et al., 2016; Visser et al., 2015; Kessler, 2012; Jiajia et al., 2016; Withycombe et al., 2022; Koleck et al., 2014). Other topics included rare diseases (7/67, 10%) (Labore, 2012; Oulton et al., 2021; Rad et al., 2022; Atienza-Carrasco et al., 2020; Laws et al., 2016; Mohammed et al., 2021; Reisinho and Gomes, 2022), pharmacogenomics (5/67, 7%) (Gleeson et al., 2013; Murray et al., 2015; Moraes et al., 2020; White et al., 2019; Henker et al., 2016), psychological or psychosocial support (4/67, 6%) (Newcomb et al., 2014; Driessnack and Gallo, 2013; Itzhaki, 2018; Mohammed et al., 2021), symptom science (4/67, 6%) (Resnick et al., 2016; Alexander et al., 2014; Voss et al., 2013; Wesmiller et al., 2014), and other (3/67, 4%) i.e., recruitment, biobanking, education, tool development (Williams et al., 2013; Newcomb et al., 2013; O'Keefe et al., 2019). Due to overlap in categories, numbers do not align exactly with content outlined in subdomains below (i.e., GC [individually]) and GC and oncology (combined).

#### 3.1.1 Sub-domain: Knowledge and understanding

**FIGURE 4:**
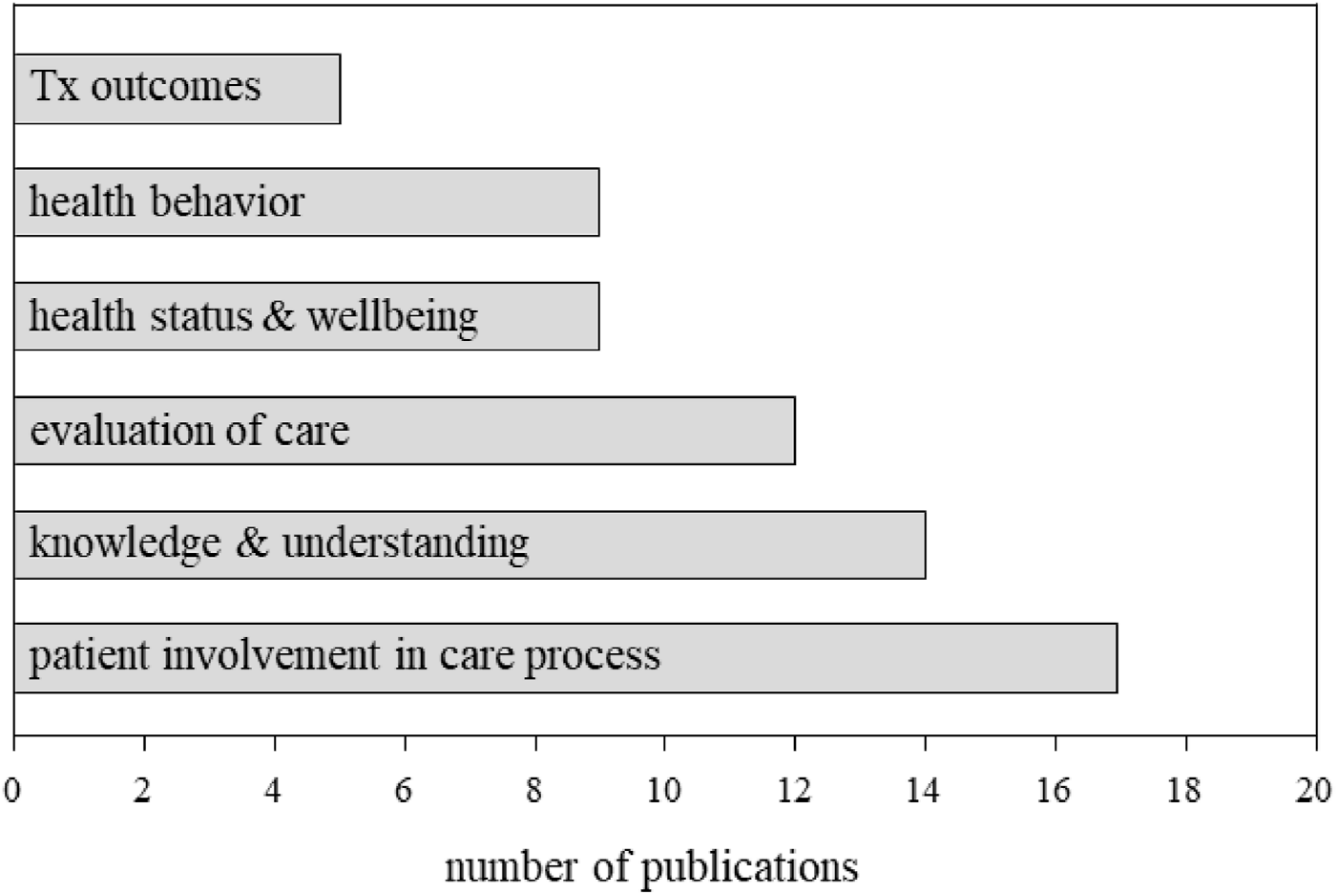
Publications across consumer-oriented outcomes sub-domains (2012–2022). Identified articles mapped to six of nine sub-domains of consumer-oriented outcomes. In total, 42/67 (63%) or articles reported on either patient involvement in the care process (i.e., decision-making), knowledge and understanding (i.e., access to information, retention, distress), or evaluation of care (i.e., satisfaction). Tx: treatment.

Fourteen (21%) articles related to the “knowledge and understanding” Cochrane sub-domain (Newcomb et al., 2019; Bracci et al., 2020; Waddell-Smith et al., 2016; Yeşilçinar and Güvenç, 2021; Almomani et al., 2020; Underwood and Kelber, 2015; Newcomb et al., 2014; Driessnack and Gallo, 2013; Zayts and Sarangi, 2013; Itzhaki, 2018; Silva et al., 2013; Gleeson et al., 2013; Underhill et al., 2012; Hamilton, 2012) (Figure 4). Studies examined various aspects related to knowledge including access and utilization (Bracci et al., 2020; Waddell-Smith et al., 2016; Almomani et al., 2020; Underwood and Kelber, 2015; Newcomb et al., 2014; Driessnack and Gallo, 2013; Zayts and Sarangi, 2013; Itzhaki, 2018; Newcomb et al., 2013; Gleeson et al., 2013), retention (Silva et al., 2013), satisfaction with information (Yeşilçinar and Güvenç, 2021), and psychological distress associated with knowledge acquisition (Underhill et al., 2012; Hamilton, 2012). Studies primarily focused on genetic counseling and screening (5/14, 36%) (Waddell-Smith et al., 2016; Yeşilçinar and Güvenç, 2021; Almomani et al., 2020; Underwood and Kelber, 2015; Zayts and Sarangi, 2013). Two articles (2/14, 14%) reported on oncogenetics (i.e., study of genes associated with inherited susceptibility for malignancy/cancer) and genetic counseling and screening (Bracci et al., 2020; Silva et al., 2013). One article (1/14, 7%) reported on pharmacogenomics and genetic counseling (Gleeson et al., 2013). While fewer studies focused on oncogenetics 2/14 (14%) (Underhill et al., 2012; Hamilton, 2012), oncogenetics and psychological/social support 1/14 (7%) (Itzhaki, 2018), psychological/social support 2/14 (14%) (Newcomb et al., 2014; Driessnack and Gallo, 2013) and “other” – tool development 1/14 (7%) (Newcomb et al., 2013). Studies employed a range of approaches that included quantitative (6/14, 43%) (Bracci et al., 2020; Waddell-Smith et al., 2016; Yeşilçinar and Güvenç, 2021; Almomani et al., 2020; Underwood and Kelber, 2015; Newcomb et al., 2013), qualitative (5/14, 36%) (Driessnack and Gallo, 2013; Zayts and Sarangi, 2013; Gleeson et al., 2013; Underhill et al., 2012; Hamilton, 2012), mixed-methods (2/14,14%) (Newcomb et al., 2014; Itzhaki, 2018), and descriptive approaches (1/14, 7%) (Silva et al., 2013).

Over half of the articles reported on non-interventional studies (8/14, 57%) (Almomani et al., 2020; Underwood and Kelber, 2015; Driessnack and Gallo, 2013; Zayts and Sarangi, 2013; Itzhaki, 2018; Gleeson et al., 2013; Underhill et al., 2012; Hamilton, 2012) while interventional studies were less frequently reported (6/14, 43%) (Newcomb et al., 2019; Bracci et al., 2020; Waddell-Smith et al., 2016; Yeşilçinar and Güvenç, 2021; Newcomb et al., 2014; Silva et al., 2013). Cumulatively articles reported that the interplay of psychological, emotional, and social impact of receiving a genetic diagnosis colored their views on decision-making and affected self-care as well as relationships. Findings highlight the importance of accessing adequate, understandable information to inform the decision-making process for genetic testing (Almomani et al., 2020; Underwood and Kelber, 2015; Itzhaki, 2018; Silva et al., 2013; Gleeson et al., 2013). Both the timing of education interventions and the methods employed (i.e., visual aids (Newcomb et al., 2014) *versus* verbal step by step instructions prior to surgery (Gleeson et al., 2013)) are critical factors to shaping decisions. Studies underscored the vital role of nurses in providing information, counseling, and support to enhance patient knowledge and high quality genetic testing decisions (i.e., informed and aligned with values and preferences) (Gleeson et al., 2013).

#### 3.1.2 Sub-domain: communication

None of the identified articles had a specific, primary focus on the subdomain of “communication” (i.e., communication aides, communication enhancement, communication skills or techniques). However, findings of articles within “evaluation of care” and “health status and wellbeing” subdomains suggest that effective communication is needed to support outcomes in the subdomains.

#### 3.1.3 Sub-domain: patient involvement in the care process

The 17 (25%) articles reporting on the Cochrane sub-domain “patient involvement in the care process” broadly examined decision making, knowledge and understanding of genetic screening and opting to participate in genetic screening (Figure 4). Within patient involvement subdomain, 9 (53%) articles (Paljevic, 2020; Newcomb et al., 2019; Li et al., 2016; Gitsels-van der Wal et al., 2015; Gitsels-van der Wal et al., 2014a; Dixon and Burton, 2014; Gitsels-van der Wal et al., 2014b; Martin et al., 2013; Hanish et al., 2018b) reported on genetic counseling and screening followed by genetic counseling and screening for cancers (3/17, 18%) (Hersperger et al., 2020; Meiser et al., 2012; Cohen and McIlvried, 2013), oncology (2/17, 12%) (Jabaley et al., 2020; Cherry et al., 2013), psychological/psychosocial support (1/17,6%) (Labore, 2012) and other (2/17, 12%) (Williams et al., 2018; Williams et al., 2013) (research recruitment and biobanking). Nearly two-thirds of articles employed a qualitative methodology (11/17, 65%) (Labore, 2012; Paljevic, 2020; Hersperger et al., 2020; Williams et al., 2018; Li et al., 2016; Gitsels-van der Wal et al., 2015; Gitsels-van der Wal et al., 2014b; Cherry et al., 2013; Williams et al., 2013; Meiser et al., 2012; Hanish et al., 2018b). Fewer studies were quantitative (4/17, 24%) (Gitsels-van der Wal et al., 2014a; Dixon and Burton, 2014; Martin et al., 2013; Cohen and McIlvried, 2013), descriptive (1/17, 6%) (Newcomb et al., 2019) along with one (6%) “other” that focused on resource development (Jabaley et al., 2020). The vast majority of “patient involvement in the care process” articles were non-interventional (13/17, 76%) (Labore, 2012; Paljevic, 2020; Hersperger et al., 2020; Newcomb et al., 2019; Williams et al., 2018; Li et al., 2016; Gitsels-van der Wal et al., 2015; Gitsels-van der Wal et al., 2014a; Dixon and Burton, 2014; Gitsels-van der Wal et al., 2014b; Cherry et al., 2013; Williams et al., 2013; Hanish et al., 2018b) and less than a quarter of articles reported findings from interventional studies (4/17, 24%) (Jabaley et al., 2020; Martin et al., 2013; Meiser et al., 2012; Cohen and McIlvried, 2013). Cumulatively, study findings point to unmet (consumer/patient) educational needs (Newcomb et al., 2019; Hanish et al., 2018a; Li et al., 2016; Martin et al., 2013; Cherry et al., 2013; Cohen and McIlvried, 2013). Highlighting a need for additional education on decision making, risks and societal and procedural aspects of genetic testing. (Martin et al., 2013). Qualitative studies primarily examined ethical considerations related to genetic testing and the decision-making process (Labore, 2012; Paljevic, 2020; Hersperger et al., 2020; Williams et al., 2018; Li et al., 2016; Gitsels-van der Wal et al., 2015; Gitsels-van der Wal et al., 2014b; Cherry et al., 2013; Williams et al., 2013; Meiser et al., 2012; Hanish et al., 2018b). Findings suggest that nursing involvement in care improved decision making, confidence and patient satisfaction (Paljevic, 2020; Dixon and Burton, 2014; Martin et al., 2013; Cohen and McIlvried, 2013).

#### 3.1.4 Sub-domain: Evaluation of care

Twelve (18%) articles related to the “evaluation of care” Cochrane sub-domain (Chandrasekaran et al., 2021; Adejumo et al., 2021; Graff et al., 2020; Murray et al., 2015; Appel and Cleiment, 2015; Oulton et al., 2021; O'Keefe et al., 2019; Rad et al., 2022; Atienza-Carrasco et al., 2020; Laws et al., 2016; Platten et al., 2012; O'Shea et al., 2012) (Figure 4). Studies broadly examined patient/participant experiences, perceptions, and satisfaction with care. Four areas of focus were identified within this subdomain including model of care 4/12 (33%) (Appel and Cleiment, 2015; Oulton et al., 2021; Rad et al., 2022; O'Shea et al., 2012), oncogenetics and genetic counseling and screening 3/12 (25%) (Chandrasekaran et al., 2021; Adejumo et al., 2021; Graff et al., 2020), genetic counseling and screening 2/12 (17%) (Atienza-Carrasco et al., 2020; Platten et al., 2012), pharmacogenomics 1/12 (8%) (Murray et al., 2015), and two “other” (2/12, 17%) (O'Keefe et al., 2019; Laws et al., 2016) (i.e., provider education and tool development). Greater than half (7/12, 58%) of articles reported on quantitative studies (Chandrasekaran et al., 2021; Adejumo et al., 2021; Graff et al., 2020; Appel and Cleiment, 2015; Rad et al., 2022; Platten et al., 2012; O'Shea et al., 2012). While fewer studies employed qualitative (2/12, 17%) (Oulton et al., 2021; Atienza-Carrasco et al., 2020), mixed-methods (1/12, 8%) (Laws et al., 2016), descriptive approach (1/12, 8%) (Murray et al., 2015), or “other” (1/12, 8%) (i.e., tool development) (O'Keefe et al., 2019). Studies were equally interventional (5/12, 42%) (Chandrasekaran et al., 2021; Adejumo et al., 2021; Appel and Cleiment, 2015; Rad et al., 2022; Platten et al., 2012) and non-interventional 5/12 (42%) (Graff et al., 2020; Oulton et al., 2021; Atienza-Carrasco et al., 2020; Laws et al., 2016; O'Shea et al., 2012). The “other” two remaining articles reported on (tool development (O'Keefe et al., 2019) and evaluation of a dedicated pediatric cardiac anticoagulation program (Murray et al., 2015)).

Findings from “evaluation of care” articles suggest nurse-led genetic services can improve patient knowledge and satisfaction with care (Chandrasekaran et al., 2021; Adejumo et al., 2021; Platten et al., 2012; O'Shea et al., 2012). However, results also indicate a need to educate healthcare providers to effectively deliver genomic information using evidence-based structured communication techniques (i.e., therapeutic education, teach-back) (Appel and Cleiment, 2015; Oulton et al., 2021; Atienza-Carrasco et al., 2020; Laws et al., 2016). In summary, articles support that nurses are well-positioned to provide genomic education and identify risk facilitating genetic testing and services.

#### 3.1.5 Sub-domain: Support

None of the identified articles had a specific, primary focus on the subdomain of “support” (i.e., practical, psychosocial).

#### 3.1.6 Sub-domain: Skills acquisition

None of the identified articles had a specific, primary focus on the subdomain of “skills acquisition” (i.e., activities of daily living, self-care, symptom control).

#### 3.1.7 Sub-domain: Health status and wellbeing

Nine (13%) studies related to the “health status and wellbeing” Cochrane sub-domain (Mohammed et al., 2021; Reisinho and Gomes, 2022; Jiajia et al., 2016; Withycombe et al., 2022; Anderson et al., 2021; Resnick et al., 2016; Koleck et al., 2014; Alexander et al., 2014; Voss et al., 2013) (Figure 4). Studies primarily focused on physical and mental health and related outcomes. Articles spanned the topics of symptom science (3/9, 33%) (Resnick et al., 2016; Alexander et al., 2014; Voss et al., 2013), oncogenetics and symptom science (2/9, 22%) (Withycombe et al., 2022; Koleck et al., 2014), and psychological/psychosocial support (2/9, 22%) (Mohammed et al., 2021; Reisinho and Gomes, 2022). One study examined genetic counseling and screening (Anderson et al., 2021) and one focused exclusively on oncogenetics (Jiajia et al., 2016). Two-thirds (6/9, 67%) of studies used a quantitative methodology (Mohammed et al., 2021; Withycombe et al., 2022; Anderson et al., 2021; Resnick et al., 2016; Koleck et al., 2014; Voss et al., 2013). Qualitative (Reisinho and Gomes, 2022), mixed-methods (Jiajia et al., 2016), and descriptive (Alexander et al., 2014) approaches were used in one study each. All (8/9, 89%) but one study (Mohammed et al., 2021) were non-interventional in nature. Studies reporting on “health status and wellbeing” outcomes highlight the importance of clinically actionable findings from genetic testing (Withycombe et al., 2022; Anderson et al., 2021; Resnick et al., 2016; Koleck et al., 2014; Alexander et al., 2014; Voss et al., 2013). Further, results emphasize the need for psychological support, prophylactic risk-reducing treatment(s), and effective communication to help ensure patients can use genetic information for making high-quality testing and treatment decisions (Mohammed et al., 2021). Articles also highlight the need for targeted interventions to educate and empower patients as well as skill development for self-management to support effective coping for living with complex, chronic conditions (e.g., cystic fibrosis) (Reisinho and Gomes, 2022). There is also a need to investigate the influence of genetic factors on treatment outcomes and quality of life (Jiajia et al., 2016).

#### 3.1.8 Sub-domain: Health behavior

Nine (13%) studies related to the “health behavior” Cochrane sub-domain (Katapodi et al., 2018; Jones et al., 2020; Salimzadeh et al., 2018; Arguello et al., 2018; Ingrand et al., 2016; Shahine et al., 2015; Visser et al., 2015; Kessler, 2012; Seven et al., 2017) (Figure 4). Studies examined aspects including patient attitudes, adherence/compliance, as well as behaviors and use of genomic interventions/services. More than half (5/9, 56%) of the articles focused on oncogenetics (Katapodi et al., 2018; Jones et al., 2020; Ingrand et al., 2016; Visser et al., 2015; Kessler, 2012) and 1/9 (11%) focused on genetic counseling/screening specifically related to oncology (Salimzadeh et al., 2018). Two articles reported on genetic counseling/screening 2/9 (22%) (Arguello et al., 2018; Seven et al., 2017) and one “other” report on provider education (Shahine et al., 2015). All nine studies employed a quantitative approach and all (8/9, 89%) but one (Seven et al., 2017) study was interventional in nature. Studies highlight the need to enhance delivery methods to improve patient access to and participation in genomic services as well as the importance of patient education (Katapodi et al., 2018; Jones et al., 2020; Seven et al., 2017). Notably, nurse-led interventions have been effective in improving patient and provider education, promoting screening behaviors, and enhancing patient outcomes (Salimzadeh et al., 2018; Arguello et al., 2018; Ingrand et al., 2016; Shahine et al., 2015; Visser et al., 2015; Kessler, 2012).

#### 3.1.9 Sub-domain: treatment outcomes

Five (8%) studies related to the “treatment outcomes” Cochrane sub-domain (Kashani et al., 2015; Moraes et al., 2020; White et al., 2019; Henker et al., 2016; Wesmiller et al., 2014) (Figure 4). Studies examined adverse outcomes as well as pathophysiological and clinical assessment factors for patients undergoing treatment. Three (60%) of articles reported on pharmacogenomics (Moraes et al., 2020; White et al., 2019; Henker et al., 2016) while symptom science (Wesmiller et al., 2014) and genetic counseling/screening (Kashani et al., 2015) were reported in the other two articles. All (4/5, 80%) but one (Henker et al., 2016) of the studies employed a quantitative approach. Similarly, all (4/5, 80%) but one (Henker et al., 2016) study were non-interventional in design. Results underscore the critical importance of systematically considering family health history for ascertaining disease risk (Kashani et al., 2015). Additionally, applying pharmacogenomics in nursing practice can help optimize medication selection/dosing, reduce adverse reactions, and increase patient satisfaction (Moraes et al., 2020; White et al., 2019; Wesmiller et al., 2014).

#### 3.1.10 *‘*Other’: theory development

One study did not fit into any of the nine “‘consumer-oriented” sub-domains (Hamilton and Kopin, 2013). The study centered on theory development in the context of oncogenetics. The qualitative study explored the lived experience of women who tested negative for hereditary breast and ovarian cancer syndrome (i.e., *BRCA1/2*). The study contributed to the refinement of the “circle of genetic vulnerability theory.”

## 4 Discussion

Our systematic scoping review of the literature (2012–2022) identified that only 29% of identified articles on genomics in nursing related to the consumer-oriented Cochrane outcome domain. A notable finding is that articles reporting consumer-oriented outcomes showed a relatively steady, linear growth in number of publications per year. This observation is interesting as one might expect that there would be an uptick in number of consumer-oriented outcomes in the published literature given the more widespread utilization of genomic healthcare and genetic testing from 2012-2022. The linear growth of publications may reflect a bias of ascertainment as it is plausible that consumer-oriented outcomes have been recorded and measured yet not published. Indeed, it is plausible that work from middle and low income countries may have been undertaken yet not published. The possibility may help explain why the identified articles were primarily from high income countries.

We identified articles relating to six of nine consumer-oriented sub-domains and one study classified as ‘other’ (theory development). None of the identified articles had a primary focus on the subdomains of ‘support’, ‘skills acquisition’, or ‘communication’. Notably, the absence of three subdomains help inform findings from other subdomains and point to future directions for therapeutic education supporting more person-centered approaches to genomic healthcare. Moreover, while all three subdomains are connected to the key nursing function of therapeutic education, they are also implicated in decision-making. Decision-making falls under the ‘Patient involvement in care’ that was the most frequently reported sub-domain (25% of identified articles). It is perhaps not surprising that decision-making has been a significant focus as genetic testing has emerged from specialty clinics into mainstream primary care. Identified articles primarily focus on challenges related to genetic testing and decision-making (Newcomb et al., 2019; Hanish et al., 2018a; Li et al., 2016; Cherry et al., 2013) (Cohen and McIlvried, 2013). Nurse-led interventions effectively improve confidence and satisfaction with genetic testing decision-making (Paljevic, 2020; Dixon and Burton, 2014; Martin et al., 2013) (Cohen and McIlvried, 2013). Overall, studies examining ‘patient involvement in the care process’ provide a deeper understanding of patients’ lived experiences and help inform more person-centered approaches to counseling that support high quality decisions (i.e., informed and aligned with values and preferences).

In light of the growing shortage of certified GCs to meet the growing demand for decisional support (Hoskovec et al., 2018; Berninger et al., 2021), there is a need for nurses and GCs to work collaboratively, and at the top of their license to meet the burgeoning demand for decisional support (Box 1). Future direction may include interprofessional studies and nurse-led interventions addressing the key need for pre-test counseling, decisional support, and “patient involvement in the care process”. One example of such patient involvement comes from a publication reporting co-creation of patient facing materials to help patients understand their genetic test results (Dwyer et al., 2021). While not identified in our literature search, this article involved nurses, GCs, physicians, and patients to co-create high-quality, patient-facing educational materials and best practices have been identified for co-creating patient-facing materials (McDonald et al., 2023).

The ‘knowledge and understanding’ subdomain was the next most common highlighting patient information gaps and unmet knowledge needs (Almomani et al., 2020; Underwood and Kelber, 2015; Itzhaki, 2018; Silva et al., 2013) - suggesting a need for more targeted educational interventions that support comprehension of the implications of genetic information and testing. Notably, these themes somewhat parallel the decision-making challenges identified in themes in the ‘patient involvement in the care process’ domain. Taken together, nearly half (46%) of all ‘Consumer-Oriented Outcomes studies’ studies coalesce high-quality decision themes, i.e., informed (knowledge deficits, information gasps) and aligned with values and preferences (i.e., decisional support). There is an opportunity for greater interprofessional collaboration (starting with interprofessional education and continuing into interdisciplinary clinical practice). The Inset Box highlights future direction for workforce development in this area as well as using technology as an additional modality to address information and knowledge gaps and for decisional support (Baroutsou et al., 2023).

Articles in the ‘evaluation of care’ subdomain reported addressing unmet patient educational needs (Murray et al., 2015; Appel and Cleiment, 2015; Rad et al., 2022) as well as increased satisfaction with nurse-led genetic counseling (Hamilton, 2012; Chandrasekaran et al., 2021; Laws et al., 2016; Platten et al., 2012) and testing support (Platten et al., 2012). It is worthwhile to note that among identified articles, nurses were primarily involved in pre-test counseling and support. Given the global shortage of GCs (Hoskovec et al., 2018; Berninger et al., 2021) and the limited uptake of cascade screening (Afaya et al., 2024), nurses could play a key role in working with patients and families to amplify cascade screening efforts and enhance equity and access to genomic healthcare thereby addressing disparities (Katapodi et al., 2023). Moreover, increased nursing involvement in pre-test counseling and decisional support could enhance healthcare delivery efficiency by enabling GCs to focus on test interpretation, post-test genetic counseling, cascade predictive familial care, giving tailored and personalized information, and support reproductive options to inform patient-led decisions. Cumulatively, data indicate that nurses can effectively deliver therapeutic education around genetic testing and are capable of providing pre-test counseling that meets the needs of patients and families. Similar to “evaluation of care”, articles reporting on the ‘health behavior’ subdomain highlighted unmet educational needs (Katapodi et al., 2018; Jones et al., 2020; Seven et al., 2017) that were amenable to nurse-led educational interventions as evidenced by increased satisfaction and screening (Salimzadeh et al., 2018; Ingrand et al., 2016; Visser et al., 2015). These data identify opportunities for synergy between nursing and GCs to help grow workforce competency in decision support and pre-test genetic counseling.

BOX 1 **Future directions for nursing and genomics relating to consumer-oriented outcomes.**

• *Global lens*: Examining and measuring consumer-oriented outcomes beyond high income and anglophone countries.
• *Patient and family engagement:* Partnering with patient and families in co-creation and co-design practices to develop more person-centered approaches to genomic healthcare.
• *Technology:* Harness large language models, artificial intelligence, and machine learning to augment decisional support with a user-centered focus.
• *Interprofessional models*: Developing, evaluating, and reporting novel interprofessional models (educational and clinical) that support competent clinicians in practicing at the ‘top’ of their licensure (scope of practice).
• *Adult learning:* Deeper examination of what modalities are most effective for closing knowledge deficits and gaps for clients.
• *Implementation into practice*: Move beyond descriptive studies to focus on developing and testing nurse-led interventional studies to improve patient-oriented outcomes.
• *Outcome measurement*: Utilize validated instruments to measure outcomes as relatively limited work has examined treatment outcomes beyond satisfaction with nurse-led interventions.
• *Consistent reporting*: Employ consistent reporting standards to facilitate transparency and comparability across studies.

It is worthwhile to note that nursing practice occurs across the continuum of care providing multiple, ongoing opportunities for individuals and families to engage with nurses across the lifespan within both inpatient hospital and ambulatory community settings. This is somewhat in contrast to encounters with genetic counselors that typically follow referral and may be single or series of encounters. As such, emphasis needs to be placed on interprofessional collaboration and developing ways to enhance the effectiveness of each aspect of an individual’s or family’s care with genetic information. A unique opportunity for cross-discipline collaboration in expanding access to genomic healthcare is the fact that nursing practice spans the entire continuum of care. As such, embedding genomic nursing competencies into nursing education (Thomas et al., 2023) can help create a workforce that can nimbly help patients and families navigate genomics healthcare across the continuum of care. Importantly, published competencies for nurses in the U.K. and United States of America have recently been updated (Calzone et al., 2024; NHS, 2023). The essentials codify the skills required to for registered nurses to provide pre-test genetic counseling and correctly select diagnostic genetic tests. Currently, the Global Genomic Nurses Alliance (G2NA) is working to establish global nursing competencies in genomics applicable to all nurses irrespective of education preparation, role, or health service design (Patch and Middleton, 2019).

The sub-domains of ‘health status and wellbeing’ include reports from nurse scientists examining the influence of genetic factors on treatment outcomes as well as health-related quality of life (Jiajia et al., 2016; Withycombe et al., 2022; Anderson et al., 2021; Resnick et al., 2016; Koleck et al., 2014; Alexander et al., 2014; Voss et al., 2013). Articles on ‘treatment outcomes’ were rather scant and tended to center on adverse reactions and how genetic testing and/or pharmacogenomics can help mitigate adverse reactions (Kashani et al., 2015; Moraes et al., 2020; Henker et al., 2016). Our validation step of including 2 GCs aimed to contextualize the findings in light of the significant focus on genetic testing/screening and decisional support/genetic counseling among the ‘Consumer-oriented’ outcome articles identified in our systematic search. The interprofessional discussion identified several key aspects including opportunities for cross-discipline collaboration and interprofessional education of healthcare professionals to develop and enhance genomic competencies.

It merits noting that most publications on GC show that there is not enough supply to meet the growing demand for genomic healthcare (Hoskovec et al., 2018; Berninger et al., 2021). While nursing and GC are distinct disciplines, there are overlaps including a shared emphasis on a person-centered approach and holistic, psychosocial support. Both nurses and GCs work across the lifespan from preconception to adult care in complementary yet different roles. The GC’s role primarily focuses on selecting/ordering the correct test given the familial context, providing accurate risk assessment, interpreting genetic variants, providing reproductive information and referrals for ongoing care. Nurses may perform aspects of genetic counseling (e.g., pre-test decisional support) in a diagnostic setting and nursing’s role concentrates on providing comprehensive, holistic across the care continuum (e.g., prior to diagnosis through treatment and providing care coordination as well as long-term follow up) for germline and somatic variants as well as pharmacogenomic results. With evolving models of care that incorporate genomics, future directions should involve closer collaboration to identify areas for the respective disciplines to function in a complementary manner while working at the top of their respective licensure. Genetic counselors supporting non-genetic specialist colleagues who are integrating genomics into their clinical practice and ensuring that ‘their knowledge and skills are appropriately translated to others’ has been advocated for some time (Patch and Middleton, 2019; Patch and Middleton, 2018).

Examples of interdisciplinary collaboration between nurses and GCs in the delivery of genomic care exist in mainstreaming programs (O'Shea et al., 2021a). Such programs initially began in cardiology (Kirk et al., 2012) as well as oncology care (O'Shea et al., 2021b) and have now expanded to other areas including renal, and ophthalmology settings where nurses and doctors are responsible for pre-test genetic counseling and consenting to genetic testing. The collaborative nature of care highlights the importance of delineating responsibility for the different aspects of the genetic counseling process and continuum (O'Shea et al., 2020). Nurses working in oncology or general care can upskill in the area of pre-test genetic counselling, consenting and test ordering for the disease context. While genetic counsellors are responsible for the post-test counselling and holistic familial care when a hereditary condition is identified or in the context of uncertain results, further genetic testing, reproductive information or when genetic risk assessments are required. As detailed in the outcomes of the studies in this review, the ongoing co-ordination of treatment, disease risk management and follow up care for the hereditary condition is in the realm of nursing care and will enhance hereditary disease survivorship and improve outcomes.

An emerging opportunity for collaboration between nurses and GCs on multidisciplinary teams is the use of exome/genome sequencing in inpatient settings, such as neonatal intensive care units (NICUs). Currently, GCs carry a large patient load providing pre- and post-test counseling, ordering tests, tracking results, and disclosing results to families. Nurses are ever present in the hospital setting yet are relatively underutilized for such tasks despite their thorough patient knowledge and relationship with families (Poston et al., 2019; Shields). Indeed, a study by Shields (Shields) highlighted that NICU nurses desire additional genetics knowledge to help increase their confidence in serving this patient population. The current scoping review suggests, genetic counsellors and nurses can work together to create resources for colleague training, offer ongoing education as new information becomes available, address ethical challenges as they arise for patients and families (e.g., secondary and/or incidental findings), and provide person- and family-centered care within the scope of their individual practices.

To ensure scalability of genomics into healthcare and improve patient-oriented outcomes, genomics must be embedded in nursing education curricula and be part of continuing professional development for practicing nurses. A comprehensive review of healthcare provider-oriented outcomes (clinical and educational) are detailed in a recent publication (Thomas et al., 2023). Educators seeking key genomics teaching resources can access them online through the International Society of Nursing in Genetics (ISONG, https://www.isong.org/ed-resources-repository) and the Inter-Society Coordinating Committee for Practitioner Education in Genomics (ISCC-PEG, https://www.genome.gov/ISCC-PEG). To integrate genomics into nursing will require an upskilling of nursing academic and continuing education faculty in genomics as well as supporting resources such as model curricula. Measuring and evaluating genomic nursing competencies will help identify areas to improve and expanding a competent nursing workforce to help realize the full potential of genomic healthcare. The process of genetic counselling is amenable to interdisciplinary collaboration with clear delineation of the practice competencies that are complementary between genetic counselors and nurses.

This special edition Research Topic has a particular focus on moving the field of human and medical genomics forward. Our scoping review highlights significant workforce challenges in meeting the growing application of genomic healthcare and precision medicine. Beyond the future directions noted in the Inset Box, broader initiatives are needed to boldly propel the field. First, re-envisioning the healthcare workforce could be a disruptive innovation for mainstreaming genomic healthcare implementation. Enhanced cross-professional dialogue and collaboration between nursing, genetic counseling and other clinicians could harmonize roles and expectations (i.e., scope of practice). For example, nurses could provide the vast majority of initial steps in the genomic healthcare continuum by identifying individuals and families who could benefit from genomic healthcare and providing information and pre-test counseling and decision support. Having nurses work at the top of their licensure and scope of practice could free GCs to focus on interpretation and subsequent healthcare decisions.

In parallel, public campaigns could support this expanded role by educating the public on nursing as integral for healthcare delivery. For example, advanced practice nurses assess, diagnose and treat (i.e., prescribe medications) and registered nurses regularly administer prescribed medications and monitor response to treatment and adverse side effects (e.g., toxicities). Thus, it is important to educate the public that professional nursing demands specialized knowledge, clinical skills, and interpersonal expertise. Increased funding of nursing research is needed to create a robust evidence base supporting best practices for person-centered genomic healthcare and decision support. Establishing best practices would not only inform improved clinical care but also help refine nursing competencies so educators and nursing schools can prepare the next-generation of nurses with required knowledge, skills and competencies to improve consumer oriented outcomes.

The emergence of artificial intelligence (AI), machine learning (ML), and large language models (LLMs) provide new opportunities to scale access to genomic healthcare. For example, chat bots leveraging LLMs could be developed using principles of human centered design to provide asynchronous decisional support thereby bridging geographic barriers to genomic healthcare for underserved communities and those living in rural or geographically remote areas. Unlike clinicians, such *in silico* approaches do not sleep. Algorithms utilizing AI/ML could be employed to run in the background of electronic health records to identify symptom clusters and family history clues to enhance detection and referral of individuals who may benefit from genomic healthcare. Such a data driven approach would also help surmount health disparities as technology lacks heuristics and implicit biases that contribute to health inequities.

## 5 Limitations

Relative strengths of the investigation include the use of a well-established framework for scoping reviews (Arksey and O'Malley, 2005; Tricco et al., 2016), the comprehensive literature search (2012–2022), use of structured search terms, rigorous dual review process, and use of Cochrane Collaboration outcome taxonomy. Several limitations merit noting. First, despite a systematic and rigorous approach, it is likely that not all articles relating to nursing in genomics were included. For example, not all articles may utilize nursing in the keywords/abstract and thus would not be retrieved in the structured search. Further, it may not be completely evident that authors involved included nurses. We did not include studies that examined nurses as the population being investigated (i.e., Nurses’ Health Study). Another limitation is that we did not conduct an extensive grey literature search so it is possible that some reports were not reviewed. Last, we did not examine risk of bias in the included articles as there was significant variability in the methodologies employed in the included studies.

## 6 Conclusion

The scoping review of consumer-oriented outcomes from nursing and/or midwifery involvement in genomics (2012–2022) identified 67 articles with a primary focus on genetic testing and screening. Synthesizing findings revealed key knowledge gaps and unmet patient informational needs around genetic testing and decision support. Moreover, consumers (i.e., patients and families) had high satisfaction with nurse-led interventions. There are opportunities for interprofessional collaboration between nursing and genetic counseling to meet the mounting demand for genomic healthcare and develop more person-centered approaches to genetic counseling and decisional support.

## Data Availability

The original contributions presented in the study are included in the article/Supplementary Material, further inquiries can be directed to the corresponding authors.
