# Supplementary material for: Consumer-oriented (patient and family) outcomes from nursing in genomics: a scoping review of the literature (2012–2022)
Source: Front Genet. 2024 Nov 29;15:1481948. doi: 10.3389/fgene.2024.1481948 (PMC11638212; doi:10.3389/fgene.2024.1481948)
Supplement: Supplementary file 1 [file Table2.docx]

Supplementary Material

# Supplementary Data S1: Search Strategy and search terms

Search terms for each respective database (PubMed, CINAHL Plus, Embase, Web of Science Core Collection).

PubMed (National Library of Medicine)

(nursing[majr] OR nurses[majr] OR nurse[tiab] OR nurses[tiab] OR nursing[tiab] OR midwife[tiab] OR midwives[tiab] OR midwifery[tiab] OR midwifery[majr] OR “nurs-ing education research”[mesh] OR “education, nursing”[mesh] OR “nursing re-search”[mesh]) AND (genes[majr] OR gene[tiab] OR genes[tiab] OR genomic*[tiab] OR genomics[majr] OR omics[tiab] OR omic[tiab] OR genetic[tiab] OR genetics[tiab] OR genetics[majr] OR “genetics, medical”[mesh] OR transcriptome*[tiab] OR tran-scriptomic*[tiab] OR "Transcriptome"[Majr] OR proteome*[tiab] OR proteomic*[tiab] OR "Proteomics"[Majr] OR "Proteome"[Majr] OR proteogenomic*[tiab] OR epigenet-ic*[tiab] OR epigenom*[tiab] OR "Epigenome"[Majr] OR "Epigenomics"[Majr] OR nu-trigenetic*[tiab] OR nutrigenom*[tiab] OR “nutritional genetic*”[tiab] OR “nutritional genomic*”[tiab] OR "Nutrigenomics"[Majr] OR metabolomic*[tiab] OR metabo-lome*[tiab] OR "Metabolomics"[Majr] OR "Metabolome"[Majr] OR pharmacogenet-ic*[tiab] OR pharmacogenomic*[tiab] OR "Pharmacogenetics"[Majr] OR microbi-ome*[tiab] OR microbiomic*[tiab] OR "Microbiota"[Majr] OR “precision healthcare”[tiab] OR “precision health care”[tiab] OR “precision medicine”[tiab] OR “precision science”[tiab] OR “personalized medicine”[tiab] OR “personalized healthcare”[tiab] OR “personalized health care”[tiab] OR “personalised medi-cine”[tiab] OR “individualized medicine”[tiab] OR “individualised medicine”[tiab] OR “personalised healthcare”[tiab] OR “personalised health care”[tiab] OR “family histo-ry”[tiab] OR “family histories”[tiab] OR “family medical history”[tiab] OR “family medical histories”[tiab] OR “symptom science”[tiab] OR symptomic*[tiab] OR "Genetic Counseling"[Mesh] OR "Precision Medicine"[Mesh]) AND (english[Filter]) AND (("2012/01/01"[Date - Publication] : "2020/12/31"[Date - Publication]))

CINAHL Plus (Ebscohost)

#1 Title: (nurse OR nurses OR nursing OR midwife OR midwives OR midwifery)

#2 Abstract: (nurse OR nurses OR nursing OR midwife OR midwives OR midwifery)

#3 Exact Subject Heading: (MH "Nurses") OR (MH "Midwifery") OR (MH "Mid-wives") OR (MH "Nursing as a Profession") OR (MH "Research, Nursing") OR (MH "Research, Midwifery") OR (MH "Nursing Science") OR (MH "Education, Nursing")

#4 #1 OR #2 OR #3 = 648,367

#5 Title: (gene OR genes OR genomic OR genomics OR omics OR omic OR genetic OR genetics OR transcriptome* OR transcriptomic* OR proteome* OR proteomic* OR epigenetic* OR epigenom* OR nutrigenetic* OR nutrigenom* OR “nutritional genetic*” OR “nutritional genomic*” OR metabolomic* OR metabolome* OR pharmacogenetic* OR pharmacogenomic* OR microbiome* OR microbiomic* OR “precision healthcare” OR “precision health care” OR “precision medicine” OR “precision science” OR “per-sonalized medicine” OR “personalized healthcare” OR “personalized health care” OR “personalised medicine” OR “personalised healthcare” OR “personalised health care” OR “individualized medicine” OR “individualised medicine” OR “family history” OR “family histories” OR “family medical history” OR “family medical histories” OR “symptom science” OR symptomic* OR symptomomic*)

#6 Abstract: (gene OR genes OR genomic OR genomics OR omics OR omic OR ge-netic OR genetics OR transcriptome* OR transcriptomic* OR proteome* OR proteo-mic* OR epigenetic* OR epigenom* OR nutrigenetic* OR nutrigenom* OR “nutritional genetic*” OR “nutritional genomic*” OR metabolomic* OR metabolome* OR phar-macogenetic* OR pharmacogenomic* OR microbiome* OR microbiomic* OR “precision healthcare” OR “precision health care” OR “precision medicine” OR “precision sci-ence” OR “personalized medicine” OR “personalized healthcare” OR “personalized health care” OR “personalised medicine” OR “personalised healthcare” OR “personal-ised health care” OR “individualized medicine” OR “individualised medicine” OR “family history” OR “family histories” OR “family medical history” OR “family medi-cal histories” OR “symptom science” OR symptomic* OR symptomomic*)

#7 Exact Subject Heading: (MH "Genetics") OR (MH "Genes") OR (MH "Genetics, Medical") OR (MH "Nutrigenomics") OR (MH "Nutrigenetics") OR (MH "Genomics") OR (MH "Pharmacogenetics") OR (MH "Genetics Nursing") OR (MH "Proteomics") OR (MH "Metabolomics") OR (MH "Proteogenomics") OR (MH "Epigenomics") OR (MH "Individualized Medicine") OR (MH "Family History")

Embase (Elsevier)

(nurse OR nurses OR nursing OR midwife OR midwives OR midwifery OR 'nursing'/exp/mj OR 'nursing research'/exp OR 'nurse'/exp/mj OR 'midwife'/exp OR 'nursing education'/exp OR 'nursing science'/exp) AND (gene OR genes OR genomic* OR omics OR omic OR genetic OR genetics OR transcriptome* OR transcriptomic* OR proteome* OR proteomic* OR proteogenomic* OR epigenetic* OR epigenom* OR nu-trigenetic* OR nutrigenom* OR “nutritional genetic*” OR “nutritional genomic*” OR metabolomic* OR metabolome* OR pharmacogenetic* OR pharmacogenomic* OR mi-crobiome* OR microbiomic* OR “precision healthcare” OR “precision health care” OR “precision medicine” OR “precision science” OR “personalized medicine” OR “per-sonalized healthcare” OR “personalized health care” OR “personalised medicine” OR “individualized medicine” OR “individualised medicine” OR “personalised healthcare” OR “personalised health care” OR “family history” OR “family histories” OR “family medical history” OR “family medical histories” OR “symptom science” OR symptomic* OR symptomomic* OR 'gene'/exp/mj OR 'genetics'/exp/mj OR 'ge-nomics'/exp/mj OR 'medical genetics'/exp OR 'omics'/exp OR 'transcriptomics'/exp/mj OR 'transcriptome'/exp OR 'metabolomics'/exp/mj OR 'metabolome'/exp OR 'prote-omics'/exp/mj OR 'proteome'/exp OR 'epigenetics'/exp/mj OR 'epigenome'/exp OR 'nu-trigenomics'/exp/mj OR 'pharmacogenetics'/exp/mj OR 'pharmacogenomics'/exp/mj OR 'personalized medicine'/exp OR 'genetic counseling'/exp OR 'genetic counselor'/exp)

Web of Science: Core Collection (Clarivate Analytics)

TS=((nurse OR nurses OR nursing OR midwife OR midwives OR midwifery) AND (gene OR genes OR genomic* OR omics OR omic OR genetic OR genetics OR tran-scriptome* OR transcriptomic* OR proteome* OR proteomic* OR proteogenomic* OR epigenetic* OR epigenom* OR nutrigenetic* OR nutrigenom* OR “nutritional genetic*” OR “nutritional genomic*” OR metabolomic* OR metabolome* OR pharmacogenetic* OR pharmacogenomic* OR microbiome* OR microbiomic* OR “precision healthcare” OR “precision health care” OR “precision medicine” OR “precision science” OR “per-sonalized medicine” OR “personalized healthcare” OR “personalized health care” OR “personalised medicine” OR “individualized medicine” OR “individualised medicine” OR “personalised healthcare” OR “personalised health care” OR “family history” OR “family histories” OR “family medical history” OR “family medical histories” OR “symptom science” OR symptomic* OR symptomomic*))

# Supplementary Data S2: Cochrane Collaboration outcome taxonomy

Three Cochrane Collaboration outcome taxonomy domains (underlined), respective sub-domains (numbered), and dimensions (bulleted). This scoping review reports on the “Consumer-Oriented Outcomes” domain.

Domain: Consumer Oriented Outcomes

1. Sub-domain: Knowledge and Understanding

- information access and use
- knowledge acquisition (i.e., level of knowledge or increased knowledge)
- retention of information, ability to recall information
- patient satisfaction with the information provided (see Satisfaction)
- psychological stress due to receiving information (see Psychological health)

2. Sub-domain: Communication

- communication aides
- communication enhancement
- communication skills or techniques

3. Sub-domain: Patient Involvement in Care Process

- decision‐making
- patient‐held information

4. Sub-domain: Evaluation of Care

- consumer‐professional interactions experience
- perceptions and ratings of care or interventions
- satisfaction

5. Sub-domain: Support

- practical support
- psychosocial support

6. Sub-domain: Skills Acquisition

- activities of daily living skills
- communication skills or techniques
- self‐care skills
- social skills
- symptom control skills

7. Sub-domain: Health Status and Wellbeing

- physical health (patient or carer)
- psychological health (patient or carer)
- psychosocial outcomes

8. Sub-domain: Health Behavior

- attitudes
- compliance / adherence
- health-enhancing lifestyle or behavior outcomes
- risk‐taking behavior
- use of interventions or services

9. Sub-domain: Treatment Outcomes

- adverse outcomes
- clinical assessments (e.g., wound healing, symptom resolution)
- pain assessment or control
- physiological measures (e.g., blood pressure, blood glucose level)

Domain: Health Service Delivery Oriented Outcomes

1. Sub-domain: Service Delivery Level

- adverse events
- health economic outcomes (e.g., costs, service utilization)
- service utilization (e.g. admission, length of stay, readmission)

2. Sub-domain: Related to Research

- involvement in research
- recruitment and retention to trials
- feedback from participation in trials

3. Sub-domain: Societal or Governmental

- health care monitoring (e.g., audit, accreditation, quality of care)
- health care planning (e.g., priority setting, policy, legislation)

Domain: Healthcare Provider Oriented Outcomes

1. Sub-domain: Knowledge and Understanding

- attitudes, behavior of health professionals
- level of knowledge or skills

2. Sub-domain: Consultation process

- practice style (e.g., patient-centeredness)
- provision of interventions

Domain: Health Service Delivery Oriented Outcomes

1. Sub-domain: Service Delivery Level

- adverse events
- health economic outcomes (e.g., costs, service utilization)
- service utilization (e.g. admission, length of stay, readmission)

2. Sub-domain: Related to Research

- involvement in research
- recruitment and retention to trials
- feedback from participation in trials

3. Sub-domain: Societal or Governmental

- health care monitoring (e.g., audit, accreditation, quality of care)
- health care planning (e.g., priority setting, policy, legislation)
